# Sarcomatoid Variant of Urothelial Carcinoma (SV-UC) of the Renal Pelvis: A Rare Tumor

**DOI:** 10.7759/cureus.83512

**Published:** 2025-05-05

**Authors:** Hussain A Alkatheri, Fatimah A Alawami, Ghadir Almajid, Zainab Almutleeg, Zainab Alabduljabbar, Dunya Alfaraj

**Affiliations:** 1 Medicine, Imam Abdulrahman Bin Faisal University, Dammam, SAU; 2 Emergency Medicine, Imam Abdulrahman Bin Faisal University, Dammam, SAU; 3 Medicine, Dar Al Uloom University, Riyadh, SAU; 4 Medicine, Dar Al Uloom University, Dammam, SAU; 5 Emergency Department, King Fahad University Hospital, Dammam, SAU

**Keywords:** biphasic tumor, case report, metastasis, renal pelvis, sarcomatoid urothelial carcinoma

## Abstract

Sarcomatoid variant of urothelial carcinoma (SV-UC) is an uncommon and highly aggressive tumor with biphasic malignant epithelial and mesenchymal differentiation. Accounting for less than one percent of all urothelial carcinomas, most cases originate in the bladder but may also involve the renal pelvis and ureters. Clinically, SV-UC resembles other urothelial carcinomas with symptoms such as macroscopic hematuria, fatigue, weight loss, and urinary obstruction. SV-UC is often diagnosed at an advanced stage with metastases and a poor prognosis, presenting a diagnostic challenge due to its similarities with other pseudosarcomatous lesions. In this case, we report a 54-year-old male with hypertension who presented with a painless, rapidly enlarging abdominal mass initially suspected to be renal cell carcinoma. Imaging revealed an atrophic right kidney with renal calculi, cortical thinning, and cystic degeneration. The patient underwent nephrectomy, and histopathology confirmed SV-UC of the renal pelvis with mesenchymal differentiation, likely metastatic to the liver and lungs. This case highlights the diagnostic and therapeutic challenges posed by SV-UC of the renal pelvis. More research is necessary to develop standardized treatment protocols for improved outcomes in this rare but aggressive malignancy with a poor prognosis.

## Introduction

Sarcomatoid variant of urothelial carcinoma (SV-UC) is a highly invasive tumor that is biphasic in nature with both malignant epithelial and mesenchymal differentiation [1]. The majority of these tumors originate from the urinary bladder, although they can also affect the renal pelvis and ureters in rare cases [2]. SV-UC in the renal pelvis is an extremely rare tumor. Thus, the current understanding of treatment and survival outcomes is largely derived from case reports [3]. In this case report, we report a 54-year-old male with a rapidly growing right-sided abdominal mass, initially suspected to be renal cell carcinoma. However, further investigation revealed a diagnosis of SV-UC of the renal pelvis, complicated by possible distant metastases to the liver and lungs. This case report highlights the complexities in diagnosing SV-UC and underscores the challenges associated with management given the aggressive nature of this tumor.

## Case presentation

A 54-year-old Filipino male, with a history of hypertension treated with amlodipine and perindopril, presented to the emergency department in April 2024. He reported a two-week history of a hard, painless, rapidly growing right-sided abdominal mass. Six months prior to this presentation, the patient experienced a solitary episode of hematuria. He denied any history of other urinary symptoms, abdominal pain, nausea, vomiting, changes in bowel habits, fever, weight loss, or night sweats. Family history revealed both parents had hypertension but no significant history of malignancy. Socially, he did not smoke but consumed alcohol occasionally.

Upon physical examination, the patient appeared well, exhibiting no signs of pain or distress. Blood pressure measured at 160/99 mmHg. Other vital signs included temperature measured at 37°C, oxygen saturation at 99%, and heart rate at 86 bpm. Abdominal examination revealed a symmetrical contour with a hard right-sided abdominal mass extending to the right flank and periumbilical region; this mass was nontender and exhibited no rigidity. There were no signs of peripheral edema or clinical evidence of deep vein thrombosis. Systemic examination, including a genitourinary assessment, was unremarkable. A bedside ultrasound demonstrated a large right-sided abdominal cystic mass with multilocular septations and hyperechoic foci, without any free abdominal fluid present.

Initial laboratory investigations revealed the following results (see Table 1). Urine cytology indicated atypical urothelial cells per The Paris System for Reporting Urinary Cytology. High suspicion for malignancy was noted, leading to a referral to urology to rule out urothelial carcinoma of the renal pelvis.

**Table 1:** Laboratory investigations

Test	Parameter	Measured Value	Units	Reference Range
Blood work	Hemoglobin	14.9	mg/dL	13.0 – 18.0
	RBC	5.43	x 10^6^/uL	4.70 – 6.10
	WBC	9.3	x 10^3^/uL	4.0 – 11.03
	Platelets	300.0	x 10^3^/uL	140.0-450.0
	Neutrophil	62.5	%	40.0 – 75.0
	Lymphocyte	22.4	%	20.0 – 45.0
	Creatinine	1.07	mg/dL	0.60 – 1.30
	Blood urea nitrogen	20.0	mg/dL	8.4 – 25.7
Urinalysis	Appearance	Light turbid amber	-	Clear, pale, yellow, amber
	Turbidity	Clear, Light	-	Light turbid
	Protein	2+	-	Negative
	Blood	>100 RBCs	-	0-3
	Leukocytes	20-30 WBCs	-	0-2
	Nitrite	Negative	-	Negative

**Figure 1:**
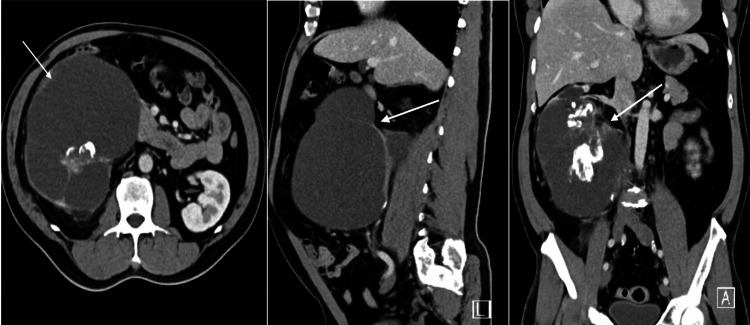
CT scan of abdomen and pelvis with contrast CT scan images showed a large right multilocular cystic mass (white arrows) in axial, sagittal, and coronal planes.

During follow-up with urology, computed tomography (CT) of the abdomen and pelvis with contrast enhancement revealed an atrophic right kidney with significant cortical thinning of the entire renal cortex and multiple renal calculi, including a 2.6 x 2.5 cm staghorn calculus in the renal pelvis. There was significant dilatation of the renal calyces with a multiloculated appearance. A notable multiloculated cyst in the lower pole measured 15.7 x 15.1 x 19 cm, containing multiple internal septations (some thickened at 8 mm) and a large internal calcification measuring 4.5 x 4 cm, which caused compression of the inferior vena cava without visualized thrombus. The right kidney's overall size was 19 cm, while the left kidney appeared unremarkable. The liver was normal in size, with a single 3 cm hypodense lesion likely representing a hepatic cyst. No abdominal free air, significant lymphadenopathy, or abnormal findings in abdominal organs were noted. The radiological findings were highly suggestive of xanthogranulomatous pyelonephritis, though cystic renal cell carcinoma could not be excluded (see Figure 1). Therefore, surgical excision was indicated for a definitive histopathological diagnosis. 

The patient was scheduled for a right open nephrectomy at the end of April 2024; however, the procedure was postponed due to poorly controlled hypertension, with preoperative blood pressures consistently above 190/117 mmHg. The patient was referred to a cardiologist to optimize his antihypertensive regimen, The patient’s blood pressure stabilized on medications, and in August 2024, he underwent an uneventful right open nephrectomy under general anesthesia.

The nephrectomy specimen weighed 1.6 kg and measured 19 x 13 x 10 cm. Gross examination revealed a distorted kidney due to cystic degeneration, primarily in the lower pole. Multiple tan-gray stones were noted within the pelvicalyceal system, with the largest measuring 4.5 x 4 x 2 cm. A well-circumscribed solid mass with cystic components, measuring 9 x 8 x 5 cm, was identified in the upper pole. Histological examination revealed a sarcomatoid variant of urothelial carcinoma (SV-UC) of the renal pelvis with focal squamous and glandular differentiation, associated with a high-grade mesenchymal neoplasm containing osseous and cartilaginous elements, spindle cell components, and osteoclast-like multinucleated giant cells. Immunohistochemistry showed focal positivity for cytokeratin 5/6 (CK5/6) and GATA-3 binding protein, supporting the diagnosis of sarcomatoid differentiation of the urothelial carcinoma. Cytogenetic studies, specifically *MDM2* gene amplification testing, returned negative results. The tumor was less than 1 mm from the renal capsular margin, but the ureteric and vascular margins were unidentifiable.

Postoperatively, the patient showed good recovery. Creatinine levels initially rose to 1.93 mg/dL and blood urea nitrogen (BUN to 27 mg/dL, but subsequently improved, with creatinine reducing to 1.5 mg/dL. the patient remained hemodynamically stable throughout his hospital stay and was discharged in good condition, with a follow-up plan for further investigations and management. A follow-up contrast enhancement CT done one month after the surgery of abdomen and pelvis indicated minimal soft tissue thickening likely related to post-surgical changes however, the possibility of underlying residual disease should be considered, and new hepatic lesions, largest was 1.1 cm in maximum axial dimension. CT of the chest with contrast enhancement identified bilateral parenchymal nodules, the largest measuring 9 x 12 cm in the right upper lobe and 8 x 11 cm in the left lower lobe, suggestive of distant metastasis. Patient was referred to oncology for systemic treatment, but unfortunately lost follow up.

## Discussion

Sarcomatoid variant of urothelial carcinoma is a rare and highly aggressive tumor, representing less than 0.3% of all other cases of urothelial carcinomas [4]. While it can originate from various sites, most cases are found in the urinary bladder. SV-UC in the renal pelvis is an extremely rare tumor, and our understanding is primarily based on individual case reports [3]. The first to report SV-UC in the renal pelvis was Piscioli et al. in 1984, and since then, few reports have documented similar findings [5].

The pathogenesis of sarcomatoid carcinoma is not fully understood. There are two suggested theories in the published literature. The monoclonal and multiclonal theory. The monoclonal theory suggests that both carcinomatous and sarcomatous tumor cells originate from a single pluripotent stem cell, which then undergoes cell differentiation into both tumor cell types. In contrast, the multiclonal theory suggests that sarcomatoid carcinoma is the result of a collision tumor composed of two or more stem cell derivatives of epithelial and mesenchymal origin [6].

The presenting symptoms of SV-UC are usually hematuria, lumbar pain on the affected side, fever, abdominal pain, general discomfort, and urinary tract irritations [7]. In this case, the patient presented with a painless, rapidly growing abdominal mass and hematuria. The diagnosis of SV-UC is challenging due to its similarities in imaging, morphology, and immunohistochemistry with other lesions showing a pseudosarcomatous pattern. A precise diagnosis requires careful morphological evaluation combined with immunohistochemical analysis to confirm its biphasic nature, characterized by both epithelial and mesenchymal differentiation [8]. In our case, the histological examination revealed a focal squamous and glandular differentiation, associated with a high-grade mesenchymal neoplasm containing osseous and cartilaginous elements. This biphasic morphology is key in distinguishing SV-UC from other differential diagnoses. Immunohistochemistry was also employed in the diagnosis, revealing both epithelial and mesenchymal tumor components, confirming the diagnosis of a sarcomatoid variant of urothelial carcinoma.

In the published literature, there is no established standard treatment for sarcomatoid variant urothelial carcinoma (SV-UC). The most commonly recommended approach involves radical surgery combined with chemotherapy and radiation therapy [9]. Recent evidence also suggests a promising role for molecular targeted therapy, as studies have shown that the majority of SV-UC cases in the upper urinary tract express epidermal growth factor receptor (EGFR), indicating that targeted therapies may offer a viable treatment option in the future [10]. However, the prognosis for SV-UC remains poor in most reported cases, with high rates of recurrence and metastasis [11].

## Conclusions

This case report underscores the diagnostic complexity and rarity of SV-UC in the renal pelvis, emphasizing its aggressive clinical behavior and poor prognosis. Continued research is essential to deepen understanding of these uncommon tumors and to develop standardized treatment protocols that may improve patient outcomes.

## References

[1] Cheng L, Zhang S, Alexander R (2011). Sarcomatoid carcinoma of the urinary bladder: the final common pathway of urothelial carcinoma dedifferentiation. Am J Surg Pathol.

[2] Moch H, Amin MB, Berney DM (2022). The 2022 World Health Organization classification of tumours of the urinary system and male genital organs-part A: renal, penile, and testicular tumours. Eur Urol.

[3] Mohan BP, Jayalakshmy P, Letha V, Bhat S (2017). Sarcomatoid carcinoma of renal pelvis involving ureter and renal parenchyma with heterologous osteosarcomatous differentiation: a case report and review of literature. Iran J Pathol.

[4] Nasrollahi H, Ahmed F, Eslahi A (2022). Sarcomatoid variant of urothelial carcinoma in the renal pelvis with brain metastasis: a case report. Pan Afr Med J.

[5] Piscioli F, Bondi A, Scappini P, Luciani L (1984). 'True' sarcomatoid carcinoma of the renal pelvis. First case report with immunocytochemical study. Eur Urol.

[6] Ahn HI, Sim J, Han H, Kim H, Yi K (2013). A case report of a sarcomatoid carcinoma arising in the renal pelvis with exuberant osteosarcomatous element. Open J Pathol.

[7] Chu Y, Ning H, Yin K (2023). Case report: sarcomatoid urothelial carcinoma of the renal pelvis masquerading as a renal abscess. Front Oncol.

[8] Nangia A, Sehgal S (2021). Multifocal sarcomatoid urothelial carcinoma of the renal pelvicalyceal system and ureter: a diagnostic dilemma. J Med Soc.

[9] Chalasani V, Chin JL, Izawa JI (2009). Histologic variants of urothelial bladder cancer and nonurothelial histology in bladder cancer. Can Urol Assoc J.

[10] Wang X, MacLennan GT, Zhang S (2009). Sarcomatoid carcinoma of the upper urinary tract: clinical outcome and molecular characterization. Hum Pathol.

[11] Tian X, Zhao J, Wang Y, Xing N (2014). Sarcomatoid carcinoma of the renal pelvis: a case report. Oncol Lett.

